# Vehicle Autonomy to Ecosystem Intelligence: A Systematic Review of Dynamic Vision Architectures in Surface Mining Operations

**DOI:** 10.3390/s26134258

**Published:** 2026-07-04

**Authors:** Nana Yaa Damtewaa Anti, Samuel Frimpong, Muhammad Azeem Raza

**Affiliations:** Department of Mining and Explosives Engineering, Missouri University of Science and Technology, Rolla, MO 65409, USA; frimpong@mst.edu (S.F.); maraza@mst.edu (M.A.R.)

**Keywords:** autonomous haulage systems, dynamic vision, surface mining, cooperative perception, digital twin, sensor fusion, ecosystem intelligence, open-pit, perception

## Abstract

Autonomous Haulage Systems (AHS) have significantly transformed surface mining operations by improving safety, productivity, and operational consistency. Currently, AHS predominantly rely on vehicle-centric perception architectures. Onboard LiDAR, radar, cameras, and Global Navigation Satellite Systems (GNSS) perform sensing, interpretation, and decision-making within individual systems. These processes enable collision avoidance and path tracking. However, they are limited in their ability to consider the broader, dynamic mining environment characterized by dust, terrain degradation, geotechnical instability, heterogeneous traffic, and rapidly evolving operational conditions. This paper presents a systematic review of dynamic vision systems of AHS in surface mining. It critically analyzes the transition from autonomy to interconnected, ecosystem-aware intelligence. The review synthesizes literature from mining automation, robotics, intelligent transportation systems, and multi-agent perception. It assesses sensing technologies, perception algorithms, sensor fusion strategies, and environmental robustness techniques. Attention is focused on the limitations of egocentric perception models in complex surface mining ecosystems. Building on identified gaps, the paper proposes a conceptual framework for Ecosystem-Centric Dynamic Vision (ECDV). Perception is enhanced through integration with fleet communication networks, dispatch systems, digital twins, geotechnical monitoring platforms, and environmental sensing infrastructure. The framework outlines a multi-layer architecture enabling cooperative perception, predictive hazard modeling, and risk-aware decision support at the mine-wide level. The review concludes by outlining a research agenda to transition from vehicle autonomy to ecosystem intelligence in surface mining. It highlights opportunities in cooperative perception, adaptive sensor fusion under degraded visibility, and digital-twin-integrated predictive safety systems.

## 1. Introduction

Surface mining occupies a uniquely hazardous position among industrial sectors. It is characterized by the interaction of large-scale mobile equipment, complex operating conditions, and the persistent exposure of personnel to high-risk environments. The combination of heavy-haul trucks, monotonous work cycles, and challenging terrain introduces fatigue, human error, and the potential for accidents. This makes operational failures both severe and often irreversible [1,2]. The emergence of Autonomous Haulage Systems (AHS) represents one of the most significant technological transitions in modern mining. This transition is driven by the need to eliminate the “human factor,” improve safety, and enhance productivity. AHS are continuously evolving and are gaining popularity for haulage solutions in surface mining. The AHS deployment in the late 2000s increased utilization, extended operating hours, and improved performance of up to 20% compared to conventional systems [2,3]. Modern AHS integrates advanced perception, control systems, communication networks, and intelligent decision-making to enable safer and more efficient haulage operations [1,3]. Leading original equipment manufacturers, such as Komatsu and Caterpillar, have fielded proprietary AHS platforms that are now operating at industrial scale across surface mining sites on multiple continents [2].

Despite these achievements, fundamental architectural limitations persist. Perception in the current AHS is predominantly egocentric. Contemporary systems maintain situational awareness through onboard sensors such as LiDAR, radar, and cameras, supported by GNSS positioning and fleet management software for routing and scheduling [4,5]. This vehicle-centric standard is proven effective within carefully delineated operational design domains.

Modern commercial mining AHS integrates LiDAR, radar, GNSS, fleet management, and obstacle detection into highly reliable production systems. However, these systems remain largely vehicle-centric. They rely on onboard sensing and make limited use of external environmental information.

Urban and highway autonomous driving have made strides in perception and object detection in the recent past. Benchmark datasets such as KITTI [6] and the Waymo Open Dataset [7] established standardized protocols for evaluating LiDAR- and camera-based object detection, tracking, and scene understanding. Similarly, the single-stage detector architecture YOLO [8] is widely used in many onboard object-detection systems that are being adapted for haul trucks, as presented in the mining-specific YOLO variants reviewed in Section 3 [9,10]. However, these benchmarks were developed for structured autonomous driving, and their underlying assumptions of consistent road geometry, defined lane structures, and comparatively predictable traffic behavior do not transfer directly to the unstructured, dust-laden, and geotechnically dynamic conditions of open-pit mines. Consequently, research on mining AHS focuses on adapting these architectures through domain adaptation, transfer learning, and mining-specific datasets rather than applying them directly. This gap between general-purpose autonomous-driving perception research and the operational realities of surface mining motivates much of the mining-specific adaptation work reviewed in this paper.

The challenge is fundamentally both perceptual and contextual. The surface mining environment presents some of the most difficult operating conditions for autonomous systems from a sensing standpoint. Dust clouds generated by haul trucks operating over unconsolidated material significantly degrade LiDAR performance. This introduces significant noise into point cloud data and drastically reduces the detection range, often by more than half in extreme cases [11,12]. Vision-based sensors are similarly constrained, as they are highly sensitive to illumination variability. This includes glare from direct sunlight, low-light conditions at night, and motion-induced image distortion caused by continuous vehicle vibration [13,14,15]. Collectively, these factors create persistent visibility degradation scenarios where current egocentric sensor fusion approaches struggle to mitigate effectively.

At a broader level, conventional vehicle-centric perception frameworks conceptualize each autonomous unit as an independent agent that relies exclusively on its onboard sensing systems for navigation and safety-critical decision-making. This inherently localized perception paradigm creates significant blind spots throughout the operational environment. Sensor range, line of sight, and environmental conditions constrain system awareness. Hazards such as slope instability, localized ground weakening, or the presence of manned equipment may exist beyond the perceptual horizon of a single vehicle. These limitations are recognized in autonomous systems, where perception is limited to immediate sensing and affected by the environment. This leads to incomplete awareness [4,16,17]. As a result, autonomous haulage operations often require supplementary input such as externally defined operational constraints or supervisory control to compensate for gaps in perception. This underscores a fundamental limitation of current vehicle-centric autonomy paradigms.

Similar limitations have been found in the field of intelligent transportation systems, where complex, real-world environments have shown that vehicle-based autonomy is insufficient. In response, research has advanced toward cooperative perception frameworks such as Vehicle-to-Everything (V2X), enabling vehicles and infrastructure to exchange sensory and contextual data and thereby extending situational awareness beyond the limits of individual sensing [18,19,20]. Simultaneously, the advent of digital twins and real-time geotechnical monitoring in mining contexts demonstrates the capacity to incorporate extensive environmental intelligence into operational decision-making [21,22]. In industrial practice, these ecosystem-level capabilities are already partially instantiated: commercial fleet management platforms such as Wenco Fleet Management System and Modular Mining’s dispatch coordinate truck–shovel assignment and routing across the mine [23,24,25]. Wenco’s Fleet Management System provides real-time fleet dispatching, equipment tracking, production monitoring, and traffic coordination. This illustrates how operational intelligence already exists at the fleet level even though it remains largely disconnected from onboard perception. Ground-based slope-stability radar systems, such as those supplied by GroundProbe, have become a standard component of open-pit geotechnical monitoring programs [26,27,28]. GroundProbe radar continuously measures slope deformation and provides early warnings of potential failures. However, these alerts are typically delivered to geotechnical personnel rather than directly integrated into autonomous haulage perception systems, illustrating the disconnect between environmental monitoring and vehicle autonomy.

In this review, ecosystem intelligence refers to a perception and decision-making paradigm that extends beyond vehicle-centric sensing by integrating information from multiple mine-wide sources, including connected vehicles, fleet management systems, fixed infrastructure sensors, digital twins, geotechnical monitoring platforms, and environmental sensing networks. By combining vehicle-level perception with ecosystem-level information, ecosystem intelligence enables a more comprehensive and context-aware understanding of the operational environment. This supports safer and more adaptive autonomous mining operations. Despite the availability of these technologies, their integration into a unified perception architecture remains under-developed.

This paper systematically reviews the gap between general-purpose autonomous driving and mining-specific adaptations, and the intelligent ecosystem for perception and decision-making for the mining-specific AHS. This paper is structured as follows. Section 2 describes the systematic review methodology. Section 3 reviews dynamic vision architecture in detail. Section 4 and Section 5 examine vehicle-level autonomy and fleet-scale ecosystem integration, respectively. Section 6 presents the ECDV framework. Section 7 addresses open challenges and emerging research directions. Section 8 and Section 9 present the discussion and conclusions, respectively.

## 2. Methodology

### 2.1. Review Design and Protocol Registration

This systematic review follows the PRISMA 2020 (Preferred Reporting Items for Systematic Reviews and Meta-Analyses) guidelines [29]. The review protocol was prospectively registered with OSF before screening began and specified the research questions, search strategy, eligibility criteria, data extraction fields, and quality assessment procedure in advance of data collection. The review protocol was designed to synthesize evidence on dynamic vision architectures deployed and evaluated in surface-mining autonomy contexts and to identify the architectural gap between current egocentric implementations and ecosystem-level intelligence requirements.

### 2.2. Search Protocol

Searches were conducted across five electronic databases: IEEE Xplore, Scopus, Web of Science, ACM Digital Library, and Google Scholar, covering the period from January 2010 to 2026. The 2010 lower bound was selected to capture the first generation of deep learning-enabled perception systems while excluding pre-deep-learning literature that is methodologically distinct. The search was structured around three concept clusters combined with Boolean operators:

Cluster A (domain): “surface mining” OR “open pit” OR “open cut” OR “AHS” OR “autonomous haulage”

Cluster B (technology): “perception” OR “vision” OR “LiDAR” OR “sensor fusion” OR “deep learning” OR “object detection” OR “semantic segmentation”

Cluster C (systems scope): “autonomous” OR “autonomy” OR “cooperative perception” OR “fleet management” OR “ecosystem” OR “digital twin” OR “V2X”

The full search string applied was (Cluster A) AND (Cluster B) AND (Cluster C). For instance, the Scopus implementation was: ((“surface mining” OR “open pit” OR “open-cut” OR “AHS” OR “autonomous haulage”) AND (“perception” OR “vision” OR “LiDAR” OR “sensor fusion” OR “deep learning” OR “object detection” OR “semantic segmentation”) AND (“autonomous” OR “autonomy” OR “cooperative perception” OR “fleet management” OR “ecosystem” OR “digital twin” OR “V2X”)) AND PUBYEAR > 2009. The IEEE Xplore and Web of Science strings followed the same Boolean logic, substituted into each platform’s field-tag syntax. Google Scholar, which does not support nested Boolean field queries, was searched using the simplified phrase combination “autonomous haulage” OR “open pit” AND (“perception” OR “LiDAR” OR “sensor fusion”) AND (“cooperative” OR “digital twin” OR “V2X”), with the first 200 results screened per query.

### 2.3. Inclusion and Exclusion Criteria

Studies were included if they;

(i) addressed sensing, perception, or decision-making for autonomous or semi-autonomous vehicles or systems in surface mining environments;
(ii) documented evidence of fleet management, cooperative perception, digital twin, or geotechnical monitoring integration with AHS systems;
(iii) reported original empirical results from field deployments, test-track trials, hardware system designs, or simulation experiments validated against real sensor data or hardware testing;
(iv) were published in peer-reviewed journals, major conference proceedings (IEEE ICRA, IROS, ITSC, Mine Automation), or substantive industry technical reports from named OEMs and research institutes; and were available in English.

Studies were excluded if they addressed exclusively underground mining, considered only teleoperation without autonomous perception, reported purely theoretical modeling without validation, or appeared only as abstracts or extended abstracts. Supplementary searches were conducted on the integration of geotechnical monitoring, digital twin applications in mining, and V2X cooperative perception.

### 2.4. Screening and Data Extraction

**Figure 1:**
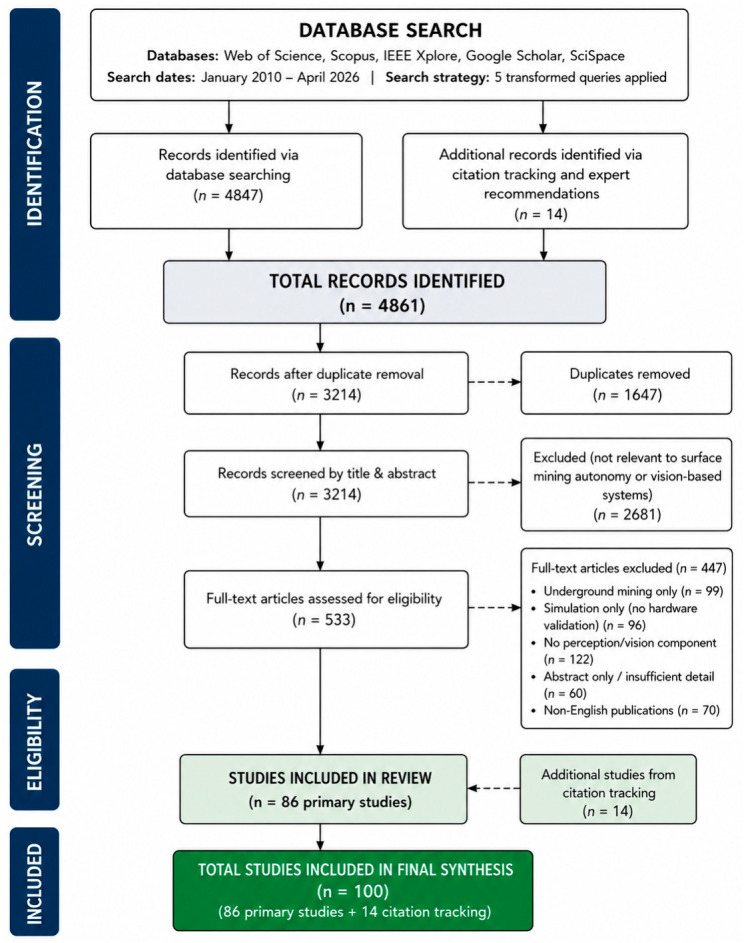
PRISMA 2020 flow diagram illustrating the study selection process for systematic review.

Title and abstract screening were conducted independently by two reviewers. Full-text screening was applied, and the inclusion and exclusion criteria were applied to the remaining papers, again independently by the same two reviewers. Disagreements at either stage were resolved through discussion between the two reviewers; cases that could not be resolved by consensus were adjudicated. Data extraction captured the architecture family, sensor modality, validation environment (field, test track, simulation), reported performance metrics, operational scale, safety outcomes, and explicitly stated limitations. The PRISMA flow diagram is shown in Figure 1.

Figure 1 presents the PRISMA 2020 flow diagram illustrating the systematic screening and selection process used in this review. A total of 4847 records were initially identified through database searching, and an additional 14 studies were identified through citation tracking and expert recommendations. These additional studies were also included in the screening process together with the database derived records. After duplicate removal, 3214 unique records remained for title and abstract screening. During this stage, 2681 records were excluded because they were not directly relevant to surface mining autonomy or vision-based systems. Consequently, 533 full-text articles were assessed for eligibility. Following the application of the predefined inclusion and exclusion criteria, 100 studies were ultimately included in the final review. The 14 supplementary studies were considered alongside all other records throughout the screening and eligibility assessment process, and eligible studies from this group were included in the final set of 100 studies. Following a detailed evaluation, 447 studies were excluded for several reasons. These were studies focused exclusively on underground mining (*n* = 99), simulation-only studies without hardware validation (*n* = 96), studies lacking a perception or vision component (*n* = 122), conference abstracts without sufficient technical detail (*n* = 60), and non-English publications (*n* = 70). Ultimately, 86 primary studies satisfied the inclusion criteria and were retained for the review. Together with 14 supplementary studies identified through citation tracking, a total of 100 studies were included in the final synthesis. The selection process ensured that the review focused specifically on validated research on autonomous haulage, perception systems, and ecosystem-level intelligence in surface mining environments.

### 2.5. Quality Assessment

Each included source was scored using a five-criterion quality-assessment rubric designed for this review, covering publication quality, methodological transparency, empirical or algorithmic validation, relevance to the review objectives, and completeness of reporting, as shown in Table 1. Each criterion was scored 1 if satisfied and 0 if not sufficiently satisfied by the same two reviewers responsible for screening, with disagreements resolved through discussion. The maximum possible score is 5. Total scores were mapped to four reliability levels: High (4–5), Moderate (3), Low (1–2), and No Reliability (0), as defined in Table 2.

Sources rated Low Reliability or No Reliability were retained for narrative and contextual discussion only and were excluded from the quantitative synthesis tables to ensure that comparative performance figures are not anchored to weakly substantiated results. Sources rated Moderate Reliability were retained in the synthesis tables but flagged; accordingly, sources rated High Reliability were treated as the primary evidentiary basis for quantitative claims. For sources retained in the synthesis tables, the reliability rating directly determined the evidence label applied. Studies validated on physical hardware at an operational or test-track mine site were labeled “deployment-validated”, whereas studies validated only in simulation, on automotive benchmark datasets, or at small research-prototype scale were labeled “research-prototype”.

## 3. Vehicle-Centric Dynamic Vision Architectures in Surface Mining

The technical architectures, performance metrics, and identified limitations are evaluated for dynamic vision systems within surface mining contexts. This section is organized across four thematic areas. This includes single-frame perception, sensor fusion, temporal and sequential perception, and edge deployment. The findings draw on 100 included studies identified through the PRISMA-compliant review process.

### 3.1. Single-Frame Perception Systems

#### 3.1.1. Two-Dimensional Object Detection

Two-dimensional object detection from monocular and stereo cameras remains the dominant visual perception primitive in current AHS. Most deployments use region-based detectors, such as Faster R-CNN or single-stage YOLO-family networks. This is because they provide a balance between accuracy and real-time performance on embedded platforms [9,30]. In automotive and road-driving benchmarks such as KITTI, Waymo Open Dataset, and BDD100K. Modern one-stage detectors such as YOLO v3–v8 typically achieve mAP values in the 70–90% range at IoU 0.5 while operating near real time, making them attractive for onboard mining hardware where compute is constrained [30,31,32]. It should be noted that the mAP figures cited are drawn from road-domain benchmarks and have not been replicated under operational conditions in surface mining.

However, evidence from surface mining analogs highlights substantial performance degradation under adverse visibility conditions. A YOLO v5-based detector required extensive architectural modifications and specialized Mine ExDark data to reach 71.9% mAP at 0.5. This outperformed the baseline YOLOv5 by 4.4 percentage points while still reflecting the domain’s difficulty [33]. More broadly, comparative studies consistently show that two-stage detectors such as Faster R-CNN retain an advantage in accuracy and minority-class robustness. This is especially true for small or partially occluded objects. In contrast, YOLO variants provide superior speed and are therefore preferred when strict real-time constraints are dominant, including in industrial and vehicular applications [9,30,34].

Across this literature, a recurrent limitation is domain shift. This is because most training still relies on generic automotive or urban datasets. Meanwhile, the visual statistics of operational mines, such as dust plumes, headlamps, sparse background structure, and different object classes and scales, are poorly represented. This leads to reduced generalization without additional adaptation [33,35].

Recent domain adaptive YOLO frameworks combine synthetic target-style data, semi-supervised labeling, and feature-level adaptation. These approaches show that much of this gap can be closed with limited labeled target data. This underscores that the main bottleneck for robust 2D perception in mining AHS is the lack of large, publicly available, labeled datasets from real open-pit operations rather than fundamental deficiencies in current detector architectures [35,36].

#### 3.1.2. Three-Dimensional LiDAR Object Detection

**Figure 2:**
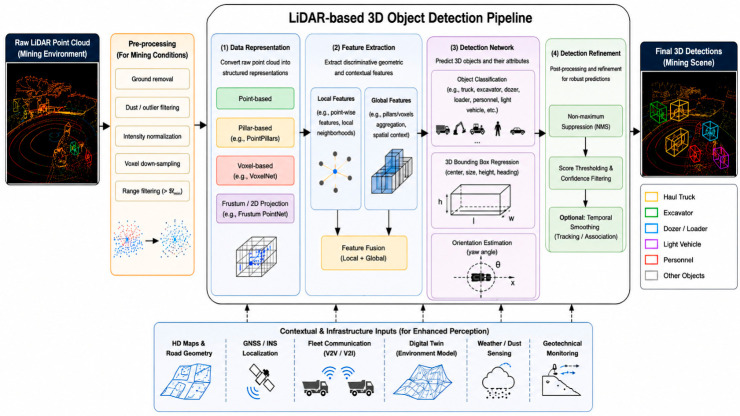
Workflow of a LiDAR-based 3D object detection system. Raw point clouds are first transformed into structured representations such as point-, pillar-, voxel-, or frustum-based formats. Geometric and contextual features are subsequently extracted and processed by detection networks to perform object classification, three-dimensional bounding-box regression, and orientation estimation. In mining environments, additional preprocessing and filtering stages are often required to mitigate the effects of dust-induced returns, point-cloud sparsity, and long-range sensing challenges.

Three-dimensional LiDAR-based object detection serves as the primary safety critical perception modality in deployed AHS. Owing to LiDAR’s robustness to ambient lighting conditions and its capacity to directly provide three-dimensional metric structure. Mining-focused adaptations of automotive 3D detectors, such as PointPillars, applied to hybrid solid-state LiDAR, achieve nearly 90% vehicle recognition accuracy under open-pit mine conditions [10]. Semantic geometric fusion pipelines developed specifically for mining datasets achieve processing rates of approximately 51 ms per frame within operational real-time latency requirements [35]. Nevertheless, point-cloud sparsity with range and the small physical size of pedestrians remain key challenges for reliable detection at long stopping distances [37]. Dust further degrades performance in ways that differ from simple occlusion. Controlled experiments show that airborne particulates produce systematic foreground returns. Frequency grows with optical depth, affecting ranging once transmittance drops below roughly 71–74% [38]. In mining and off-road settings, this has motivated the development of dedicated dust-filtering algorithms that exploit LiDAR intensity and local spatial structure to remove sparse dust points while preserving obstacles [11]. Learning-based and RGB–LiDAR fusion approaches have also been developed to improve dust classification and F1 scores over conventional filters [39]. Together, these studies indicate that current voxel-based or pillar-based architecture can meet real-time latency budgets on embedded hardware. However, maintaining safe detection performance in dusty, long-range mining environments requires explicit modeling and filtering of dust-induced returns rather than relying on clean-weather automotive training. Figure 2 illustrates the general workflow of LiDAR-based 3D object detection systems commonly adopted in autonomous driving and adapted for autonomous haulage applications. Raw point clouds are first transformed into structured representations such as point-, pillar-, voxel-, or frustum-based formats. Geometric and contextual features are subsequently extracted and processed by detection networks to perform object classification, three-dimensional bounding-box regression, and orientation estimation. In mining environments, additional preprocessing and filtering stages are often required to mitigate the effects of dust-induced returns, point-cloud sparsity, and long-range sensing challenges. Figure 2 depicts the typical workflow of LiDAR-based 3D object detection systems, which are commonly used in autonomous driving and are tailored for autonomous haulage. The process begins by converting raw point clouds into structured formats such as points, pillars, voxels, or frustums. Then, geometric and contextual features are extracted and fed into detection networks for tasks such as object classification, 3D bounding-box regression, and orientation estimation. In mining settings, additional preprocessing and filtering steps are often necessary to address challenges such as dust interference, sparse point clouds, and long-distance sensing issues.

#### 3.1.3. Radar-Based Perception

Radar occupies a distinct position in the AHS sensing stack because its operating wavelength is one to two orders of magnitude longer than that of LiDAR or visible-light cameras. Automotive radar units typically operate at 24 GHz or 77–79 GHz (corresponding to wavelengths of approximately 12.5 mm and 3.8–4 mm, respectively), while emerging 4D imaging radar for mining applications has been demonstrated in the 76–81 GHz band. Airborne dust particles in surface mining are predominantly in the PM10/PM2.5 range, which is several hundred times smaller than the radar wavelength. Because scattering efficiency falls sharply when particle diameter is much smaller than the wavelength, mmWave radar signals pass through dust clouds with comparatively little attenuation, in contrast to LiDAR and visible-light cameras, whose wavelengths are close to or larger than the dust particle size and are therefore subject to significant scattering and backscatter [38,40].

Field studies support this physical expectation. Mining-focused evaluations of 4D mmWave radar report that the technology enhances autonomous haulage systems’ ability to detect obscured static obstacles at dumping sites, where dust concentrations peak during tipping operations [41]. Controlled comparisons of LiDAR and 77 GHz imaging radar mounted side-by-side on operational haul trucks during extended field trials found that radar measurement count and detection range remained stable across dust-affected shifts, with no major degradation observed. In contrast, LiDAR performance is known to deteriorate [42]. A related line of work generating controlled, multi-level dust concentrations to study mmWave propagation found that while dust introduces ghost detections and multipath interference, these effects can be substantially mitigated through threshold-based filtering on radar-specific parameters such as radar cross-section, Doppler velocity, and angular position, rather than through the signal attenuation mechanisms that dominate LiDAR and camera degradation [43].

Beyond raw penetration, 4D radar’s Doppler dimension provides direct velocity measurement for every detected point, which is unavailable from a single LiDAR or camera frame without temporal differencing. This is reported to enable earlier warning of fast-approaching vehicles or personnel under degraded visibility than frame-based sensing alone [44]. The principal limitations of radar relative to LiDAR and camera systems are its angular resolution, which remains coarser even in 4D imaging variants, and a relative scarcity of mining-specific validation datasets, since most existing radar perception literature originates from automotive road contexts.

#### 3.1.4. Ultrasonic Sensors

Although cameras, LiDAR, and radar dominate perception architectures for AHS, ultrasonic sensors, a form of active acoustic sensing, provide complementary capabilities for short-range obstacle detection in degraded visibility conditions. Unlike passive acoustic systems that detect naturally occurring sounds, ultrasonic sensors actively emit high-frequency sound waves, typically between 20 and 40 kHz. It estimates object distance by measuring the time-of-flight of reflected signals. Their effective sensing range is generally limited to a few centimeters up to approximately 5 m, making them suitable for low-speed collision avoidance, blind-spot monitoring, docking assistance, and close-proximity maneuvering rather than the long-range perception required during haulage operations [45].

Unlike optical sensors, active ultrasonic sensing is minimally affected by airborne dust because acoustic wavelengths at typical operating frequencies are several orders of magnitude larger than respirable mining dust particles. Consequently, dust produces negligible attenuation of ultrasonic signals [46] effective detection range under dusty conditions [47,48]. This complementary behavior makes ultrasonic sensing particularly valuable during periods of reduced visibility, where optical perception may be temporarily degraded.

Despite these advantages, ultrasonic sensing has several limitations that restrict its use as a standalone perception modality in autonomous haulage. Detection range is inherently short, angular resolution is lower than that of LiDAR and imaging radar, and measurement accuracy can be affected by highly irregular or sound-absorbing surfaces. Consequently, ultrasonic sensing is best deployed as a complementary modality that enhances, rather than replaces, LiDAR and radar within multimodal perception architectures. Within AHS perception architectures, ultrasonic sensors are particularly suited to loading, dumping, parking, and other low-speed maneuvers where accurate close-range obstacle detection is essential for safe operation [49].

Although ultrasonic sensing is widely adopted in robotics and autonomous vehicles, mining-specific validation remains limited. Few studies have evaluated its performance under the combined effects of heavy dust, severe vibration, large vehicle dimensions, and elevated ambient noise characteristic of surface mining environments.

### 3.2. Sensor Fusion Architectures

**Figure 3:**
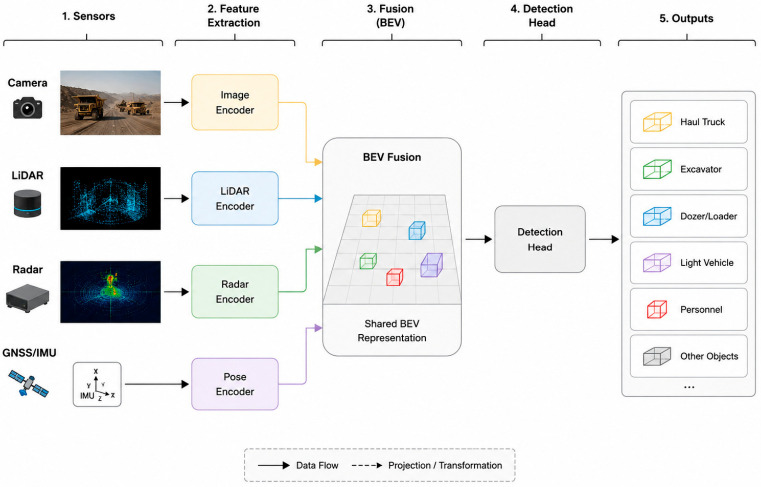
Multi-sensor features fusion framework for surface mining perception. Camera, LiDAR, radar, and GNSS/IMU data are encoded into complementary feature representations and fused within a shared bird’s-eye-view (BEV) space. The fused representation supports robust detection and tracking of mining assets, vehicles, personnel, and environmental obstacles in a complex operational environment.

Multi-sensor fusion in mining leverages the complementary strengths of cameras, LiDAR, radar, GNSS, and IMU to achieve robust perception and localization under dust, vibration, and long-range visibility requirements. Classical surveys of road vehicles define the standard fusion taxonomy as high-, low-, and mid-level fusion [5]. Modern autonomous haulage systems rely on the complementary strengths of multiple sensing modalities to achieve robust perception under challenging mining conditions. Figure 3 illustrates a generalized feature-level sensor fusion architecture in which information from cameras, LiDAR, radar, and positioning sensors is transformed into a common representation and fused to support object detection and environmental understanding. Recent deep-learning reviews further refine this into BEV-centric fusion and cross-modal attention paradigms. This highlights unified BEV grids as an effective common space for integrating heterogeneous modalities [50]. BEV fusion exemplifies this trend by projecting camera and LiDAR into a shared BEV representation. This preserves both semantic richness and geometric accuracy while remaining computationally efficient for multi-task perception [50]. In mining-specific settings, PV Fusion shows that perspective-view fusion with depth densification and attentional feature fusion can better support >200 m perception in surface mines than conventional BEV models tuned for urban ranges [51]. Robustness to dust and adverse weather increasingly motivates radar integration. 4D mmWave radar is identified as a key upgrade for “Mining 5.0,” offering richer Doppler and elevation information than 3D radars and improving autonomy in surface mining operations [52]. Practical surface mining deployments demonstrate LiDAR–radar fusion with adaptive confidence re-weighting to filter dust and stably detect 30–40 cm obstacles at 60 m on unpaved roads [53]. Broader mmWave radar vision fusion reviews emphasize data, feature, and decision-level schemes, along with demanding calibration requirements [54]. Across all architectures, accurate, preferably online, multi-sensor calibration and synchronization are repeatedly highlighted as foundational to any reliable fusion system in autonomous vehicles and mining trucks [5]. The BEV-centric fusion architecture discussed has been validated primarily on road autonomous driving benchmarks such as nuScenes and Waymo. Their application to surface mining remains at the adaptation and prototype stage, with PV Fusion representing the most mining-specific implementation identified in this review.

### 3.3. Temporal and Sequential Perception

Temporal and sequential perception focuses on understanding how a scene evolves. These systems can track moving agents, estimate changing surface conditions, and anticipate rare but dangerous events. In mining-like settings, this includes predicting the future positions of personnel and vehicles, monitoring haul-road degradation via tire tracks, and detecting anomalous motions such as spoil pile collapses or bench failures. Methods from autonomous driving and robotics offer directly transferable tools for these tasks. Optical flow captures pixel-level or point-level motion between frames and underpins motion understanding and tracking. Recurrent and temporal fusion networks, such as Long Short-Term Memory networks, Gated Recurrent Units, and temporal transformers, integrate sequences of accelerometer, camera, or LiDAR data. This is to improve state estimation and classification over single-frame approaches. This is shown for traffic flow prediction, video action recognition, accident anticipation, and multi-object tracking [55,56]. The models exploit temporal context to better handle occlusions, noise, and complex interactions between multiple moving agents. 4D spatiotemporal occupancy and sequence-based 3D detection extend 3D perception with an explicit time dimension. Architectures such as OccFormer and RenderOcc represent scenes as voxelized 3D occupancy with semantics. Spatiotemporal networks predict future occupancy grids several seconds ahead without explicit object tracking [57]. Object-centric temporal detectors and trackers propagate object queries or proposals over time to model motion and interactions efficiently, thereby improving accuracy and robustness in dynamic scenes [33]. These 4D-style approaches align with the concept of “four-dimensional spatiotemporal detection” described. They are well-suited to forecasting the propagation of bench failures or haul-road degradation once trained on mining-specific data. The temporal and sequential methods reviewed in this section are drawn from autonomous driving and robotics literature. Their transferability to surface mining is conceptually well-founded but has not yet been empirically demonstrated using mining-specific datasets or AHS deployments.

### 3.4. Edge Deployment and Real-Time Inference

Real-time perception for autonomous haul trucks must meet strict end-to-end latency requirements while running on sealed, vibration-tolerant embedded platforms. Across vision, LiDAR, and vision-language pipelines, work on Jetson/AGX Orin-class devices shows that meeting ~20–100 ms per-frame budgets is feasible only with aggressive, hardware-aware compression and optimization of perception models [31,58]. To fit within the onboard, compute budgets while preserving safety-relevant accuracy, deployments combine structured pruning, post-training quantization, and knowledge distillation. Surveys highlight these as the core compression tools for edge deployment, often used jointly [59,60]. INT8 PTQ tailored for LiDAR achieves up to ~3× speedup with almost no accuracy loss on CenterPoint, directly targeting edge devices. Mixed-precision PointPillars with TensorRT achieves up to a 2.5× latency reduction compared to FP32, while fully integer PTQ for PointPillars maintains FP32-level accuracy and enables low-latency hardware acceleration. For PointPillars-like 3D detectors, FPGA implementations with 8/2-bit hybrid quantization achieve ~15.6 FPS while maintaining acceptable detection quality [61].

Knowledge distillation further narrows the gap between compact students and heavier teachers: structured KD for 3D detection can compress PointPillars 4× while improving mAP over the teacher, and KD frameworks for 3D detectors reduce FLOPs by more than half while preserving or surpassing teacher accuracy and achieving >2× runtime speedup on high-end GPUs [62]. Combined schemes such as PQK co-optimize pruning, quantization, and KD to produce lightweight models explicitly aimed at constrained devices [33]. Collectively, these results support deploying compressed 2D, 3D, and open-vocabulary perception models on Orin-class platforms within tight latency envelopes, with only modest accuracy trade-offs when compression is carefully designed and calibrated [61]. To provide a structured overview of the perception approaches identified in the reviewed literature, Table 3 summarizes the major dynamic vision architecture families by sensor modality, representative models, validation environments, reported performance metrics, and mining-specific limitations. The table highlights both the maturity of existing perception technologies and the challenges associated with their deployment in surface mining environments.

## 4. Vehicle Autonomy and Perception Challenges in Surface Mining

### 4.1. Vehicle Autonomy in Surface Mining: Current State

AHS represents a widely deployed form of vehicle autonomy in surface mining. Major original equipment manufacturers, particularly Komatsu and Caterpillar, have successfully deployed autonomous fleets across large-scale iron ore, copper, coal, and oil sands operations. These systems operate within tightly controlled operational design domains (ODDs) that include geofenced mine boundaries, pre-surveyed haul roads, and centralized fleet management systems. Continuous monitoring from remote operation centers enables reliable production with minimal human intervention [63].

Current AHS architecture remains fundamentally egocentric. Each vehicle relies primarily on onboard LiDAR, radar, cameras, and GNSS to perceive and interpret its surroundings. While this approach performs effectively within structured environments, situational awareness remains limited to the vehicle’s local sensing horizon. As mine operations become larger, deeper, and more complex, this localized perception model increasingly struggles to account for dynamic environmental conditions, mixed-traffic interactions, and hazards beyond direct line of sight. The limitations of vehicle-centric autonomy become more apparent when considering the broader range of autonomous mining equipment now emerging in the industry. Autonomous drills require highly accurate positioning under severe GNSS multipath interference near pit walls, while autonomous dozers depend on continuous terrain reconstruction and real-time blade load estimation. Auxiliary vehicles such as water trucks, fuel bowsers, and grade-control vehicles introduce additional complexity because they operate with unpredictable trajectories and frequently interact with both autonomous and manned equipment in mixed-traffic environments. These operational realities highlight an important distinction between mining autonomy and conventional road vehicle automation. Although current AHS are commonly categorized as SAE Level 4 systems, mining autonomy depends less on increasing vehicle-level automation and more on improving the information architecture supporting situational awareness [3,64]. The transition toward ecosystem intelligence, therefore, represents a shift from isolated onboard perception toward distributed, mine-wide awareness supported by fleet management systems, cooperative perception, infrastructure sensing, and shared environmental intelligence.

### 4.2. Environmental Perception Challenges

The reviewed studies consistently identify six environmental factors that degrade the performance of onboard perception systems in AHS. These challenges arise from the interaction between harsh mining environments and the physical operating principles of different sensing technologies, making an understanding of these mechanisms essential for selecting appropriate sensor modalities and designing robust multi-sensor fusion architectures.

Dust generated during blasting, loading, and haulage operations is one of the most significant obstacles to reliable perception. Because suspended dust particles are comparable in size to the wavelength of visible light, cameras and LiDAR experience significant scattering and attenuation, resulting in reduced image contrast and degraded ranging performance. Dust accumulation on sensor surfaces further degrades sensing quality, while dehazing techniques provide only partial recovery under dense dust conditions [65,66]. In contrast, millimeter-wave radar is considerably less affected because dust particles scatter its longer wavelength minimally.

Post-blast conditions further degrade perception through the combined effects of suspended dust, combustion gases, and thermal gradients, which distort optical propagation and create rapidly changing visibility conditions. These effects primarily affect cameras and LiDAR, whereas radar maintains more stable performance under adverse atmospheric conditions [67].

Localization is similarly challenging in deep open-pit mines, where limited satellite visibility and multipath propagation degrade GNSS accuracy and positioning reliability. Although machine learning and three-dimensional map-assisted localization techniques have shown promise for mitigating these effects, validation in operational mining environments remains limited [68,69].

Vision-based perception is also affected by the extreme illumination contrasts created by reflective pit walls and deep shadows, which often exceed the dynamic range of conventional cameras. While event-based cameras offer improved performance under high-dynamic-range conditions, their application in surface mining remains limited [70]. In addition, continuous vibration from rough haul roads degrades MEMS-based inertial measurement units, reducing localization accuracy, particularly when GNSS performance is simultaneously compromised [68].

Perception is frequently constrained by occlusions from large mining equipment, stockpiles, and infrastructure, particularly in mixed-traffic environments. Although cooperative perception via V2X communication has the potential to extend situational awareness beyond line-of-sight, field-validated implementations in surface mining remain scarce [17].

Collectively, these environmental challenges demonstrate that no single sensing modality can provide reliable perception across all operating conditions encountered in surface mining. Instead, the complementary characteristics of optical sensors, radar, inertial sensors, and positioning technologies reinforce the need for robust multi-sensor fusion architectures. Table 4 summarizes these environmental challenges, their impacts on individual sensing modalities, current mitigation strategies, and the remaining research gaps identified in the literature.

## 5. Fleet Intelligence and Ecosystem Integration

This section synthesizes evidence from the reviewed literature on ecosystem-level data sources that complement onboard vehicle perception. Fleet management systems, cooperative perception networks via V2X communication, fixed-infrastructure sensing, digital twin platforms, and geotechnical monitoring systems are increasingly mature technologies already deployed in major mining operations. This section examines the current state of each ecosystem integration layer and identifies the technical and organizational barriers to their coupling with vehicle-level perception and decision support.

### 5.1. Fleet Management Systems and Perception Coupling

Fleet management systems constitute the existing layer of mine-wide intelligence in automated surface mining operations. Fleet management platforms such as Wenco, Modular Mining’s dispatch, and Sandvik’s OptiMine are reported to aggregate GPS-derived vehicle positions, payload measurements, fuel consumption data, and maintenance alerts. This helps to optimize cycle times, reduce queue congestion, and maximize shovel utilization. However, these systems operate at a logistics and scheduling level. They do not currently consume or broadcast real-time perceptual data. AHS trucks generate LiDAR point clouds, camera feeds, and radar detections that remain siloed within each vehicle’s onboard computer platform [18,71].

This architectural separation means that safety-critical environmental information detected by one vehicle, such as an obstacle at a dump or a damaged berm section, is not shared with other vehicles approaching the same location. Each vehicle independently detects or fails to detect the same hazard. The gap between FMS-level situational awareness and vehicle-level perceptual awareness is the primary architectural bottleneck in the transition toward ecosystem intelligence.

### 5.2. Cooperative Perception and V2X in Mining

Cooperative perception refers to the sharing of sensory data or derived perception outputs between vehicles and infrastructure to construct a shared environmental model. This has advanced substantially in the field of autonomous driving. V2X architectures encompassing Vehicle-to-Vehicle (V2V), Vehicle-to-Infrastructure (V2I), and Vehicle-to-Network (V2N) communication have been standardized through IEEE 802.11p (DSRC) and C-V2X (cellular) protocols and validated in road platooning and intersection management applications [18,19].

The translation of cooperative perception to surface mining is still in its early stages. Key distinctions from the road context include the physical scale of mine operations (pit diameters of 1–10 km with significant elevation changes); the severity of the communication environment (pit-wall reflections, dust attenuation of wireless signals, and limited 5G coverage in deep pits); and the heterogeneity of the vehicle fleet (autonomous trucks, manned light vehicles, manual dozers, shovel operators, and drones). Wang et al. [33] demonstrated a cooperative perception prototype at a research mine site in China, using LTE V2X to share compressed LiDAR feature maps among two autonomous trucks and an RSU-mounted camera, achieving a 40% reduction in blind-zone area in the loading zone without exceeding available bandwidth constraints. This proof of concept represents the current frontier of cooperative perception in mining; no production deployments were identified in the reviewed literature.

The maturity of road V2X communication provides valuable insights for the evolution of cooperative perception and ecosystem-level data integration in surface mining. However, direct adoption is constrained by the unique operational characteristics of mining environments. In autonomous road vehicles, standardized V2X communication architectures have enabled interoperable V2V, V2I, V2N and V2P communication, allowing connected agents to exchange perception information, extend situational awareness beyond the sensing capabilities of individual vehicles, and support collaborative decision-making [72,73,74]. However, the evidence synthesized in this review indicates that surface mining presents additional operational requirements that are not fully addressed by existing road-oriented V2X frameworks. First, ecosystem-level perception requires the integration of heterogeneous information sources, including onboard sensors, infrastructure sensing systems, fleet management systems, digital twins, geotechnical monitoring platforms, and environmental sensing networks, rather than relying solely on vehicle-to-vehicle communication. Second, communication frameworks should accommodate the unique characteristics of open-pit mining environments, including pit-wall signal reflections, variable terrain elevations, dynamic wireless coverage, and long-range operational requirements. Third, interoperability frameworks should support heterogeneous mining fleets comprising autonomous haul trucks, manually operated vehicles, stationary infrastructure, and unmanned aerial systems with diverse sensing and communication capabilities. Collectively, these findings suggest that future mining communication frameworks can build upon the architectural principles of mature V2X ecosystems while developing mining-specific extensions that facilitate interoperable ecosystem-level data integration, cooperative perception, and coordinated autonomous operations across the mining enterprise.

### 5.3. Infrastructure Sensing Integration

Fixed sensing infrastructure, including cameras and radar systems mounted at dump points, shovel pedestals, pit entry ramps, and berm edges, provides a complementary perspective. It is inherently free of the dust and vibration constraints that affect vehicle-mounted sensors. Several Tier 1 mining companies have deployed fixed camera arrays for traffic management and personnel safety monitoring at high-risk locations [75]. However, these systems operate as independent safety overlays rather than as data sources integrated into the AHS perception pipeline. The technical pathway for infrastructure integration comprises two components: a communication interface between fixed sensor nodes and a central fusion server (achievable with existing LTE/5G private network infrastructure), and a data fusion architecture that combines detections from the fixed infrastructure and the vehicle ego into a coherent shared representation. The latter requires geometric calibration of fixed sensor frames to the mine coordinate system and temporal synchronization across heterogeneous hardware [76,77].

### 5.4. Digital Twin Integration

Mining companies are increasingly investing in digital platforms that integrate geospatial information, equipment telemetry, operational processes, and enterprise data to create dynamic representations of mining operations. Although many of these systems represent important precursors rather than fully realized digital twins, they provide the technological foundation for network-centric mining by supporting visualization, simulation, and enterprise-wide operational coordination [78,79]. Commercial platforms such as Hexagon HxGN, Caterpillar MineStar, and Bentley iTwin provide key integration capabilities by combining survey data, fleet telemetry, geological models, production schedules, and infrastructure information into unified operational environments. These platforms are continuously enriched by drone photogrammetry, LiDAR surveys, GNSS positioning, equipment telemetry, and enterprise databases, enabling increasingly accurate and up-to-date representations of the evolving mine environment [79].

Integrating digital twin information directly into the AHS perception pipeline represents a significant yet largely unexplored opportunity for ecosystem-level perception. Rather than relying solely on onboard sensors, AHS perception systems could exploit contextual information maintained by the digital twin. This includes current haul road conditions, geotechnical hazard zones, blast exclusion boundaries, temporary road closures, fleet locations, equipment health, and anticipated traffic patterns. This contextual knowledge extends perception beyond the vehicle’s immediate sensing horizon, allowing perception algorithms to reason about operational conditions that may not be directly observable through onboard sensing alone. Like the role of high definition maps in autonomous road vehicles, digital twin provides a continuously evolving environmental priority. However, unlike static HD maps, digital twins can reflect the rapidly changing conditions of active mining operations, including excavation progress, blasting activities, road maintenance, and evolving traffic patterns. By integrating data across information technology and operational technology systems, digital twins have the potential to improve localization robustness, perception reliability, trajectory planning, and safety-critical decision-making through mine-wide situational awareness [79].

Despite this potential, digital twins have not yet been fully integrated into real-time perception pipelines for autonomous mining vehicles. Existing digital twin implementations primarily support operational planning, visualization, simulation, and enterprise-level decision-making, in which system updates typically occur at operational timescales rather than perception timescales. In contrast, onboard AHS perception performs localization, sensor fusion, and obstacle detection within tens of milliseconds. This disparity in temporal resolution creates a fundamental systems integration challenge. Future research should therefore investigate hierarchical synchronization architectures in which only rapidly changing, safety-critical information, such as newly detected hazards, equipment positions, traffic conditions, and geotechnical alerts, is streamed directly into the perception pipeline. In contrast, less dynamic operational information is updated asynchronously. Such selective real-time synchronization would transform the digital twin from a passive operational management platform into an active source of contextual intelligence for autonomous perception, advancing the transition from vehicle-centric autonomy toward true ecosystem-level situational awareness.

### 5.5. Geotechnical Monitoring as a Perception Input

Geotechnical instability is one of the most significant safety hazards in surface mining. This is because it threatens personnel, mobile equipment, and production continuity [26]. To mitigate these risks, modern slope stability monitoring employs complementary sensing technologies. This includes ground-based slope stability radar, satellite Interferometric Synthetic Aperture Radar (InSAR), GNSS receivers, automated total stations, extensometers, inclinometers, MEMS tilt sensors, and distributed optical fiber sensing. These technologies provide complementary spatial and temporal coverage for monitoring surface deformation, displacement rates, crack propagation, and progressive rock mass instability. Recent advances further integrate AI and IoT architecture to improve automated hazard detection, predictive analysis, and multi-sensor data fusion for continuous slope assessment [26,27].

These monitoring systems continuously generate high-frequency geotechnical information describing displacement magnitude, deformation velocity, acceleration trends, and evolving failure mechanisms. Progressive acceleration in slope displacement is widely recognized as one of the most reliable precursors to impending slope failure and forms the basis of Trigger Action Response Plans (TARPs). This enables engineering inspections, evacuation procedures, and the establishment of exclusion zones before catastrophic collapse occurs [26,27]. Distributed optical fiber sensing has also demonstrated significant potential for detecting localized strain accumulation, fracture initiation, and crack evolution before visible failure occurs, while satellite InSAR complements ground-based radar by providing regional-scale deformation monitoring across multiple pits, enabling the identification of previously unknown or slowly evolving geotechnical hazards that may not be captured by localized monitoring systems [80].

Despite these advances, geotechnical monitoring systems are rarely directly coupled with the AHS perception and motion-planning pipelines. In current mining operations, deformation measurements and hazard assessments are typically communicated to geotechnical engineers or control room operators, who subsequently implement operational responses such as haul road closures, revised traffic routing, speed restrictions, or personnel exclusion zones through established operational procedures. Although this workflow provides an effective safety management process, it introduces a human-mediated delay between hazard detection and autonomous vehicle response, limiting the ability of AHS to react immediately to rapidly evolving geotechnical conditions.

Within the ECDV framework, geotechnical monitoring serves as an ecosystem-level perception layer that continuously augments onboard sensing with mine-wide environmental intelligence by integrating displacement measurements, radar alarms, InSAR deformation maps, distributed optical fiber strain measurements, and geotechnical risk assessments. This enables autonomous haulage vehicles to dynamically update maps, establish no-go zones, reroute traffic, adjust vehicle speeds, and anticipate hazards beyond the onboard sensing horizon, thereby improving operational safety and decision-making.

The principal research challenge is therefore no longer the development of additional sensing technologies, but the real-time integration of heterogeneous geotechnical information into autonomous perception and decision-making frameworks. Recent reviews emphasize that future slope stability monitoring should focus on multi-sensor integration, AI-assisted interpretation, IoT-enabled monitoring, and interoperable sensing architectures rather than isolated monitoring technologies [26]. However, direct integration of geotechnical monitoring outputs into AHS perception remains largely unexplored. Among the ecosystem-level integration opportunities identified in this review, incorporating geotechnical monitoring into autonomous perception is one of the immediately deployable approaches, as much of the sensing infrastructure already exists in modern surface mining operations. Transforming these systems from passive monitoring tools into active perception inputs would enable autonomous haulage systems to anticipate geotechnical hazards before they become observable by onboard sensors, advancing the transition from vehicle-centric autonomy toward true ecosystem-level situational awareness. Table 5 summarizes the principal integration layers identified in the literature, their current deployment maturity, their role in enabling ecosystem intelligence, and the key research gaps that must be addressed to support large-scale implementation.

## 6. Ecosystem-Centric Dynamic Vision (ECDV): A Conceptual Framework

### 6.1. Motivation and Design Principles

This paper proposes the ECDV framework, which is motivated by a specific architectural claim. The primary barrier to the next generation of AHS capability is not just vehicle-level perception accuracy. This has reached a sufficient baseline for geofenced operation. But the absence of a structured information architecture connecting vehicle perception to the broader mine system. ECDV is not a replacement for onboard perception. It is an augmentation layer that systematically addresses the blind spots of egocentric systems by integrating mine-wide information into the vehicle’s situational awareness.

The framework is grounded in four design principles. First is graceful degradation. Ecosystem-level information augments but does not replace onboard perception as the primary safety-critical input. The system must operate safely when external data sources are unavailable. Second is latency stratification. Different information sources operate on different update timescales. The architecture must route time-critical data, such as V2V cooperative detections, through low-latency paths. Slower-updating data, such as digital twin state information, must be routed through higher-latency but richer pathways. Third is uncertainty propagation. All externally sourced data carries uncertainty estimates. These estimates must be propagated through the fusion pipeline and reflected in the vehicle’s risk assessment. Fourth is explainability. To support incident investigation and regulatory audits, decisions influenced by external data must be traceable back to their source.

### 6.2. ECDV Layer Architecture

The ECDV framework comprises five functional layers arranged in a hierarchical information architecture. Table 6 summarizes each layer, including its primary components, data sources, functional responsibilities, and outputs. This layered representation illustrates how onboard perception can be progressively enriched through cooperative sensing, ecosystem context, predictive safety modeling, and human–machine interaction. Each layer consumes and enables graceful degradation when any layer is unavailable. Layer 1 (L1: Onboard Perception) encompasses the existing egocentric sensing and detection pipeline, comprising LiDAR, camera, radar, and GNSS/IMU, which produces a local occupancy map and object list at operational frame rates. This layer is unchanged from the current AHS architecture. Layer 2 (L2: Cooperative Perception) introduces external perceptual Data from V2X communication, compressed across neighboring vehicles and RSU infrastructure sensors. A cooperative BEV fusion module combines L1 and L2 data within a shared spatial reference frame. This extension increases the effective detection range and improves the resolution of occluded regions. Communication scheduling algorithms prioritize data from vehicles whose geometric positions most effectively complement the ego vehicle’s field of view. Layer 3 (L3: Ecosystem Context) integrates non-perceptual mine-system data, including the digital twin state, FMS traffic intent, geotechnical risk indices, blast schedule, and weather/dust monitoring outputs, into a risk-annotated environment model. This layer operates on timescales of seconds to minutes for most parameters, with sub-second updates for geotechnical alarm states and traffic intent signals.

**Figure 4:**
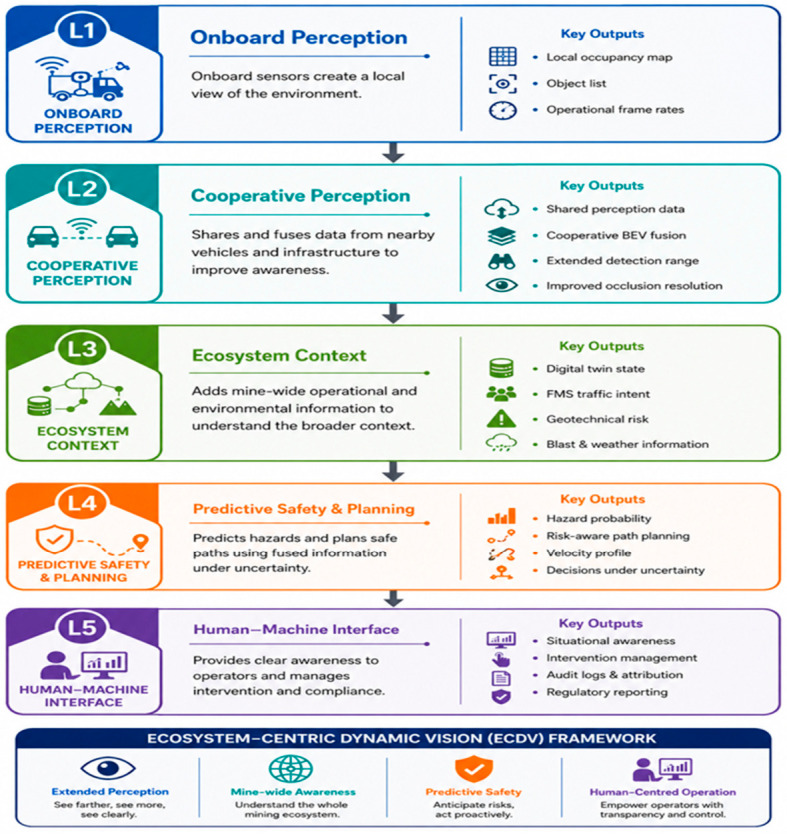
ECDV framework for surface mining operations. The framework comprises five hierarchical layers: (L1) onboard perception, (L2) cooperative perception, (L3) ecosystem context, (L4) predictive safety and planning, and (L5) human–machine interface. Together, these layers integrate vehicle-level sensing, shared perception, mine-wide contextual information, and risk-aware decision support to enable the transition from vehicle autonomy to ecosystem intelligence.

Layer 4 (L4: Predictive Safety and Planning) consumes the fused L1–L3 representation to perform predictive hazard modeling and risk-aware path planning. A probabilistic hazard model estimates the likelihood and consequences of candidate hazard scenarios, including personnel in the path, slope failure, and road washout, given current sensor evidence and ecosystem context. Risk-aware planning algorithms generate velocity profiles and path alternatives that maintain acceptable risk levels under uncertainty, including conditions where sensor data is degraded. Layer 5 (L5: Human–Machine Interface) presents synthesized situational awareness to control room operators, supports intervention request management, and maintains audit logs that attribute decisions to their data sources. This layer also manages the interface with regulatory reporting systems, as depicted in Figure 4.

## 7. Challenges and Emerging Directions

### 7.1. Open Technical Challenges

Several open challenges currently limit the transition from vehicle autonomy to ecosystem intelligence in surface mining. The absence of a mining-specific perception of benchmarks is the most consistently cited barrier across the reviewed literature. The field risks optimizing automotive benchmarks that do not reflect mining reality. Rigorous evaluation and comparison of perception architectures are impossible without large-scale, publicly available datasets that capture the full range of operational conditions. These conditions include dust, night operations, rain, vibration, and geotechnical events. Without such datasets, the field risks optimizing automotive benchmarks that do not accurately reflect the realities of mining.

Safety certification for machine learning-based perception functions poses regulatory and methodological challenges in the absence of established precedent. Functional safety standards, including IEC 61508 and the mining-specific ISO 17757 (Earth-moving Machinery Autonomous and Semi-Autonomous Machine System Safety), were developed primarily for deterministic control systems. Applying these standards to neural network-based perception, whose failure modes are difficult to characterize exhaustively. This requires developing new verification and validation methodologies that integrate formal methods, statistical testing, runtime monitoring, and operational data analysis [81].

Communication architectures for cooperative perception in deep open-pit mines present challenges that differ substantially from conventional road V2X environments. The highly dynamic mine topology, steep pit walls, frequent blasting, and continuously changing operating conditions can adversely affect wireless communication reliability, underscoring the importance of robust, low-latency communication for cooperative perception [82]. Cooperative perception further increases communication demands, as connected vehicles exchange LiDAR point clouds or intermediate perception features to extend perception beyond the sensing horizons of individual vehicles. Consequently, recent research has focused on feature compression, selective information sharing, and network-aware cooperative perception to reduce communication overhead while maintaining perception performance [83,84,85].

Recent advances in mining communication infrastructure indicate that private 5G networks, combined with MEC, provide a promising foundation for supporting data-intensive applications. Zhang et al. [76] demonstrated that integrating edge caching with dynamic resource allocation reduced average communication latency to approximately 15 ms while achieving average uplink and downlink transmission rates of approximately 1 Gbps and 1.5 Gbps, respectively, highlighting the potential of edge-assisted communication architectures for intelligent mining systems.

### 7.2. Emerging Technology Directions

Foundation models and vision-language models (VLMs) offer a potential pathway to reducing the labeled-data requirements of mining perception systems. Models such as the SAM have demonstrated strong zero-shot and few-shot segmentation capabilities across a range of computer vision applications. While these results suggest potential applicability to mining perception tasks, evidence from large-scale surface mining deployments remains limited, and further validation under mining-specific operating conditions is required [86,87,88]. Preliminary evaluations demonstrated that SAM achieved competitive performance for mining terrain segmentation in adjacent domains compared with specialized models while using only 5% of the labeled data [61]. This suggests that foundation model adaptation could substantially reduce the labeling burden for new mine sites.

Neuromorphic and event-based sensing offers a promising response to the high-vibration, high-dynamic-range challenge of the mining environment. Event cameras generate asynchronous pixel-level responses to luminance changes rather than fixed-frame images. They provide microsecond temporal resolution and a dynamic range exceeding 120 dB. This performance is substantially superior to that of frame-based cameras in direct solar-to-artificial lighting transitions encountered on mine sites [89,90]. Integration of event cameras with LiDAR has shown promise in autonomous driving and robotics research, particularly in challenging lighting and high-dynamic-range environments. However, its application to surface mining remains largely unexplored, and no mining-specific validation studies were identified in the reviewed literature. Federated learning offers a potential route to training perception models on aggregated operational data from multiple mine sites without centralizing proprietary production data. A federated training protocol, in which each site trains locally and shares only model updates while keeping raw data local, could facilitate the development of mining-specific perception models by leveraging data from multiple geographically distributed operations. In this approach, each site trains locally and shares only model weight gradients rather than raw data. This approach has demonstrated privacy-preserving capabilities with differential privacy guarantees in comparable industrial IoT contexts, though mining-specific validation remains an open research direction [91].

### 7.3. Regulatory and Standardization Pathways

The regulatory landscape for AHS continues to evolve as mining operations progress toward increasingly autonomous and interconnected systems. ISO 17757:2019 establishes the principal international framework for the safety of autonomous and semi-autonomous machine systems used in earth-moving applications. However, the standard provides limited guidance on the implementation, validation, and certification of advanced perception systems that integrate heterogeneous sensing, distributed intelligence, and cooperative decision-making [92]. Similarly, the Western Australia Work Health and Safety (Mines) Regulations 2022 [93], which provide the regulatory framework for large-scale commercial AHS deployments in Western Australia. They adopted a risk-based approach requiring mine operators to identify hazards, implement appropriate control measures, and demonstrate that risks have been reduced to as low as reasonably practicable through systematic risk management and ongoing safety assurance [93]. This performance-based regulatory approach accommodates technological innovation while requiring robust verification and validation processes to demonstrate the safety of increasingly complex autonomous systems.

Beyond regulatory requirements, the evidence synthesized in this review suggests that technological standardization is an essential enabler for the safe deployment of ecosystem-level perception architectures in surface mining. Across the reviewed studies, recurring challenges related to heterogeneous sensing, communication interoperability, environmental uncertainty, and mixed-fleet coordination highlight the need for standardized approaches to information exchange and system integration. Ge et al. [94] similarly, identify the absence of dedicated standards for intelligent mining as a major contributor to heterogeneous equipment architecture, limited interoperability, and increased implementation costs. This motivates the development of mining-specific technical specifications that cover autonomous transportation, communication infrastructure, production safety, operational management, and cooperative operations. Notably, these emerging initiatives incorporate several functional components that closely align with the ECDV framework proposed in this review, including communication infrastructure, cooperative operations, centralized operational management, and intelligent system coordination.

In contrast, the autonomous road vehicle sector has benefited from mature technological standardization through V2X communication and cooperative perception frameworks, enabling interoperable information exchange, distributed situational awareness, and coordinated decision-making among connected vehicles and infrastructure [72,73,74]. Collectively, these developments provide valuable reference models for mining-specific communication architectures, interoperability requirements, validation methodologies, and cooperative perception frameworks. Rather than directly adopting road vehicle standards, the evidence synthesized in this review suggests that mining-specific standardization can build upon these established principles while incorporating requirements unique to surface mining, including communication under complex pit geometries, perception data exchange with infrastructure-based sensing systems, heterogeneous fleet interoperability, geotechnical hazard communication, and functional safety under dynamically changing environmental conditions.

As the operational design domains of AHS continue to expand beyond isolated haul roads to encompass mixed-traffic environments, degraded visibility, dynamic weather conditions, and geotechnically unstable areas, both regulatory and technological frameworks will need to evolve in parallel. The evidence synthesized in this review indicates that future mining standards would benefit from supporting the integration of onboard perception with infrastructure sensing, fleet management systems, digital twins, geotechnical monitoring platforms, and environmental sensing networks while establishing consistent approaches for interoperability, validation, and safety assurance [74,94]. Collectively, these developments support the transition from vehicle-centric perception to ecosystem-level intelligence, providing a pathway for regulatory guidance and technological standardization to jointly enable safe, scalable, and interoperable autonomous mining systems.

## 8. Discussion

### 8.1. Synthesis of Findings

This systematic review examined the dynamic vision architectures underpinning surface-mining autonomy across the literature spanning 2010–2026, with particular emphasis on how advances in perception are evolving toward ecosystem-level intelligence through the integration of vehicle sensing, cooperative perception, fleet management systems, digital twins, and geotechnical monitoring. Three overarching findings emerge from this synthesis.

Vehicle-level perception has advanced substantially in technical maturity, but this progress has been confined to a narrow operational envelope. The reviewed deployment literature shows that AHS operating within geofenced, pre-mapped, FMS-managed environments under moderate environmental conditions achieves safety and productivity outcomes that justify large-scale commercial adoption. This is a genuine and important achievement. However, the same literature consistently shows that performance degrades sharply outside this envelope, in heavy dust, in mixed-traffic zones, near geotechnically active slopes, or when GNSS is degraded. Egocentric architecture provides no mechanism to compensate for these failures using external information sources.

The reviewed literature provides limited evidence of real-time integration among digital twins, geotechnical monitoring systems, infrastructure sensing platforms, and AHS perception layers. This suggests that ecosystem-level data integration remains an important area for future development. The reviewed literature indicates that ecosystem-level technologies are emerging as complementary research domain alongside vehicle-level perception. Although their deployment levels vary across applications, the evidence suggests they have significant potential to support the transition toward large-scale autonomous mining. This is not primarily a technology gap but an integration and standardization gap. The ECDV framework proposed in Section 6 is intended as a structured articulation of how these existing data sources could be connected to vehicle perception.

The research literature on cooperative perception, while rapidly advancing in automotive contexts, has produced only a few mining-specific contributions. The physical and operational characteristics of surface mines include the scale, depth, communication environment, and fleet heterogeneity.

### 8.2. Comparison with Adjacent Domains

The evolution of surface mining autonomy shares several similarities with developments in autonomous road driving, particularly the transition from vehicle-centric autonomy to connected and cooperative perception architectures. The trajectory of road autonomous driving: the transition from individual vehicle autonomy to infrastructure-connected cooperative intelligence. Like the autonomous driving sector, surface mining autonomy is evolving from isolated vehicle-level autonomy toward connected, cooperative systems that integrate vehicles, infrastructure, and operational data sources. The road AV industry has learned, at high cost, that Level 4 capability in geofenced domains does not naturally scale to more complex environments without qualitative architectural changes [95]. Surface mining should internalize this lesson proactively rather than encounter it reactively with greater consequences.

The parallel with precision agriculture is also instructive. Agricultural autonomy has rapidly shifted toward ecosystem-centric architectures: UAV-based field sensing feeds into ground-vehicle path planning, soil-moisture networks inform irrigation-robot decisions, and satellite imagery informs harvest timing [96,97,98]. These integration patterns are directly analogous to those proposed by the ECDV framework for mining, and the agricultural implementation experience offers practical design guidance.

### 8.3. Limitations of This Review

This review is subject to a few limitations. The predominance of automotive perception literature in the dataset, reflecting the relative scarcity of mining-specific perception publications, may introduce a bias toward architectures optimized for road environments. Operational data from AHS deployments is limited and largely commercially confidential. This restricts the ability to assess real-world performance beyond OEM-reported metrics. The proposed ECDV framework is conceptual and has not been validated in deployment.

## 9. Conclusions

This systematic review synthesized evidence from studies published between 2010 and 2026 to examine the evolution of dynamic vision architectures for AHS in surface mining. The review traced the evolution of perception systems from conventional onboard sensing to increasingly connected, context-aware architectures, drawing on the literature from mining engineering, robotics, intelligent transportation systems, and computer vision. Beyond vehicle-level sensing, the review examined the ecosystem-level data sources that operate alongside AHS. This includes fleet management systems, cooperative perception, digital twins, and geotechnical monitoring.

Three principal findings emerge from the reviewed literature. First, perception technologies have advanced substantially over the past decade. Improvements in deep learning, LiDAR-based object detection, millimeter-wave radar, temporal perception, edge computing, and multi-sensor fusion have significantly enhanced the accuracy, robustness, and computational efficiency of onboard perception systems. These advancements have supported the reliable commercial deployment of autonomous haul trucks within carefully delineated operational domains.

Second, the review identifies environmental robustness as the dominant unresolved challenge limiting autonomous perception in surface mining. Across the reviewed studies, airborne dust, post-blast atmospheric conditions, vibration, GNSS degradation, mixed-traffic interactions, terrain variability, and geotechnical instability consistently reduce the reliability of perception, regardless of sensing modality. LiDAR performance is particularly sensitive to airborne particulates, with ranging accuracy degrading as atmospheric transmittance falls [38]. Although sensor fusion improves resilience by exploiting complementary sensing characteristics, no existing perception architecture consistently maintains robust situational awareness across the diverse environmental conditions encountered in operational surface mines. These limitations reflect not only the physical constraints of individual sensors but also the inherent limitations of vehicle-centric perception architectures.

Third, the review reveals that many of the technologies needed to extend perception beyond vehicles are already in commercial use, even though they remain functionally disconnected from AHS perception pipelines. Fleet management platforms such as Wenco and Modular Mining dispatch aggregate truck position, payload, and scheduling data at a commercial scale. But they do not currently consume or broadcast the LiDAR, camera, and radar detections generated onboard each truck [18,71]. Digital twin platforms, including Hexagon HxGN, Caterpillar MineStar, and Bentley iTwin, integrate survey data, fleet telemetry, and geological models into unified operational environments, yet function at planning and visualization timescales rather than the millisecond timescales required for perception [78,79]. Geotechnical monitoring systems, including slope stability radar, satellite InSAR, and MEMS-based sensing, continuously track displacement and deformation as precursors to slope failure. However, their outputs are typically routed to control room operators rather than directly into vehicle motion planning [26,27]. Research on cooperative perception, in which vehicles and infrastructure share sensor data to construct a common environmental model, has been demonstrated in prototype form but has not reached production deployment in mining. Consequently, the principal gap identified in the literature is no longer the availability of sensing technologies. However, the absence of a unified architecture that integrates these already-mature, independently operated systems into a coherent, real-time perception framework.

This architectural gap motivates the ECDV framework proposed in Section 6. ECDV organizes this integration across five functional layers, extending from onboard perception (L1) through cooperative perception via V2X communication (L2), non-perceptual ecosystem context drawn from digital twins and geotechnical feeds (L3), predictive hazard modeling and risk-aware planning (L4), to a human–machine interface that supports operator oversight and audit (L5). Rather than replacing onboard perception, the framework augments it while preserving four safety-critical design constraints: graceful degradation when external data sources are unavailable, latency-stratified routing of time-critical and slower-updating information, propagation of uncertainty from external sources into the vehicle’s risk assessment, and explainability sufficient to support incident investigation. The framework is offered as a structured hypothesis for how the ecosystem-level technologies cataloged above could be connected to vehicle perception. It has not been implemented or validated, and its contribution at this stage is conceptual rather than empirical.

Advancing this agenda requires progress on several fronts identified in Section 7. The absence of large-scale, publicly available, mining-specific perception datasets and benchmarks remains the most consistently cited barrier in the reviewed literature. This is because it leaves the field dependent on automotive benchmarks that do not reflect mining-specific conditions such as dust, night operation, and geotechnical events. Safety certification for machine-learning-based perception also lacks an established precedent. Existing functional safety standards, including IEC 61508 and the mining-specific ISO 17757:2019, were developed primarily for deterministic control systems, and applying them to neural network-based perception will require new verification methodologies that combine formal methods, statistical testing, and runtime monitoring [81]. Communication architecture presents a further constraint. Reviewed work on feature compression and network-aware cooperative perception seeks to reduce the bandwidth burden of sharing point cloud data among vehicles [83,84,85]. Recent evidence suggests that private 5G networks, combined with mobile edge computing, can meaningfully address this constraint, with one deployment reporting average communication latency of approximately 15 milliseconds and uplink and downlink throughput of approximately 1 and 1.5 gigabits per second, respectively [76].

Several emerging technologies identified in this review offer promising, though still largely unvalidated, pathways for mining-specific perception. Foundation and vision-language models, such as the Segment Anything Model, have shown potential to reduce labeled-data requirements in adjacent domains. With one adaptation for terrain segmentation, it is reported to achieve competitive performance using only 5% of the labeled data required by specialized models. Event-based and neuromorphic sensing respond to per-pixel luminance changes with microsecond resolution and a dynamic range of over 120 dB. These characteristics suggest potential applicability to the lighting transitions and vibration encountered in surface mines, but no mining-specific validation studies were identified in the reviewed literature [89,90]. Federated learning allows perception models to be trained across multiple mine sites without centralizing proprietary operational data. While federated learning could theoretically provide substantially greater dataset diversity by enabling collaboration across multiple mining sites, it remains an area of ongoing research rather than a demonstrated capability in autonomous mining.

Regulatory and standardization pathways will need to evolve alongside these technical developments. ISO 17757:2019 establishes the principal international safety framework for autonomous earth-moving machinery. But offers limited guidance on certifying perception systems that integrate heterogeneous sensing and cooperative decision-making [92]. Jurisdictional frameworks such as the Western Australia Work Health and Safety (Mines) Regulations 2022 take a risk-based approach that accommodates innovation while requiring demonstrated risk reduction, offering a possible template for ecosystem-aware AHS certification [93]. The absence of dedicated technical standards for intelligent mining has itself been identified as a contributor to heterogeneous equipment architecture and limited interoperability across the sector [94]. As discussed in Section 8.2, the development of autonomous road vehicles serves as a warning: the road autonomy industry discovered, often at great expense, that Level 4 capabilities confined to a geofenced area cannot be expanded to more complex settings without fundamental architectural changes [95]. Surface mining is positioned to internalize that lesson proactively, through standardization and ecosystem integration. As discussed in Section 8.3, this review is subject to limitations, including the predominance of automotive perception literature in the reviewed dataset and the limited availability of non-confidential operational data from commercial AHS deployments. These constraints should be considered when interpreting the findings summarized above.

Overall, the reviewed literature indicates that vehicle-level perception technologies in surface mining have advanced considerably. Ecosystem-level perception and information integration remain comparatively underrepresented in the published literature, despite substantial advances in many of the underlying technologies. Future research should prioritize mining-specific perception datasets and benchmarks, field validation of cooperative perception architectures under operational mining conditions, safety certification methodologies suited to learning-based perception, and empirical testing of ecosystem-centric frameworks such as ECDV against real deployment data. Progress in these areas would strengthen the evidence base for the transition from vehicle autonomy to ecosystem intelligence in surface mining. This would help ensure that advances in perception algorithms are matched by equivalent progress in system integration, standardization, and operational validation.

## Figures and Tables

**Table 1:** Source quality assessment criteria and scoring framework.

Criterion	Description	Score
Publication quality	Peer-reviewed journal article, peer-reviewed conference paper, academic book by reputed publishers, or recognized technical standard/report	0–1
Methodological transparency	Provides a clear description of methods, assumptions, data sources, algorithms, and design procedures	0–1
Empirical or algorithmic validation	Includes field validation, case study evidence, numerical validation, simulation testing, or algorithmic performance evaluation	0–1
Relevance to review objectives	Directly addresses haul road layout, geometry, safety integration, AHS path planning, or haulage/operational performance	0–1
Completeness of reporting	Provides sufficient detail to support interpretation, comparison, or replication	0–1

**Table 2:** Reliability classification thresholds.

Total Score	Reliability Level	Interpretation
4–5	High Reliability	Strong methodological quality, clear relevance, and sufficient validation or reporting
3	Moderate Reliability	Useful evidence with some limitations in validation, reporting, or scope
1–2	Low Reliability	Limited validation, limited methodological detail, or non-peer-reviewed contextual source
0	No Reliability	Not relevant

**Table 3:** Taxonomy of dynamic vision architecture families reviewed by sensor modality, representative models, validation environment, key performance metrics, and mining-specific limitations.

Architecture Family	Sensor Modality	Representative Models	Validation Environment	Key Performance Metrics	Mining-Specific Limitations
2D Object Detection	Camera	YOLOv8, Faster R-CNN, SSD	Controlled test tracks; synthetic datasets	mAP 78–92% (clean)	Severe degradation under dust; night sensitivity
3D Object Detection	LiDAR	Point Pillars, CenterPoint, VoxelNet	KITTI, nuScenes (road-domain)	mAP 55–82% (3D IoU)	Point cloud sparsity > 50m; dust returns; vibration noise
Semantic Segmentation	Camera + LiDAR	Deep Lab v3+, RandLA-Net, SqueezeSegV3	Mining terrain datasets (limited)	mIoU 68–85%	Limited labeled mining data; terrain class imbalance
Multi-modal Fusion (early/mid/late)	LiDAR + Camera + Radar	BEV Fusion, Transfusion, Point Painting	Autonomous driving benchmarks	NDS 0.65–0.71	Cross-modal calibration drift; rain/dust degrades early fusion
Temporal/Sequential Modeling	LiDAR sequence, camera video	4D-Occ, BEV-Flow, ConvLSTM	Simulated mine environments	Velocity error < 0.3 m/s	Latency accumulation; no mining-specific benchmarks
Transformer-based (ViT/BEV)	Camera (multi-view)	BEV Former, DETR3D, PETR	nuScenes; road data	NDS ~0.56–0.62	Compute-intensive; unproven in dust/vibration
Edge-deployed/Compressed Models	Camera, LiDAR	Pruned YOLOv8, TensorRT-quantised PointPillars	Onboard GPU (Orin, TX2)	Latency < 50ms; 10–30% accuracy trade-off	Memory constraints limit model depth; calibration complexity

Note: Performance figures in Table 3 are sourced from the studies reviewed in Section 3. Where a study was validated on road-domain benchmarks rather than a mining site, this is indicated in the Validation Environment column; such figures should not be read as mining-validated performance.

**Table 4:** Environmental factors affecting AHS onboard perception performance, current mitigation strategies, and residual capability gaps.

Environmental Factor	Affected Sensor(s)	Observed Performance Impact	Current Mitigation Strategy	Residual Gap
Airborne Dust (PM10/PM2.5)	LiDAR, Camera, Radar	LiDAR range reduced to 75%; camera contrast degraded > 60%	Adaptive thresholding; multi-return LiDAR	No real-time dynamic compensation; no mine-specific benchmarks
Mud and Water Occlusion	Camera, LiDAR window	False positive rate elevated 3×; sensor window contamination	Compressed air cleaning; redundant cameras	No predictive contamination modeling
Direct Solar/Night Glare	Camera	Detection mAP drops ~40% in direct sun and is near zero at night without IR.	IR cameras; HDR imaging; LiDAR primary	Mixed lighting transitions are unhandled
High Vibration (haul roads)	Camera, LiDAR	Image blur; LiDAR point drift; calibration drift over hours	Shock-mounted housing; periodic recalibration	Real-time in-motion recalibration remains unsolved
GNSS Outage/Multipath (pit walls)	GNSS	Position error > 5 m; path planning failure	IMU dead reckoning; HD map prior	Prolonged outages degrade SLAM convergence
Geotechnical Instability	None (ego sensors blind)	No onboard detection; sudden bench failure	Periodic human inspection; slope radar (fixed)	No real-time integration with the AHS perception pipeline

Note: The performance-impact figures in Table 4 reflect the specific test conditions reported in the studies reviewed in Section 4.2. They are not the authors’ own estimates and should not be extrapolated beyond the environmental ranges tested in those studies.

**Table 5:** Ecosystem integration technologies: current deployment maturity, contribution to ecosystem intelligence, and key research gaps identified in the reviewed literature.

Integration Layer	Technology/Platform	Current Deployment Maturity	Contribution to Ecosystem Intelligence	Key Research Gaps
Fleet Management Systems	Wenco, Modular Mining, Dispatch	Commercially mature; deployed at major AHS operations	Route optimization, traffic conflict resolution, shift scheduling	No real-time perceptual feedback loops from trucks to FMS
V2X/Wireless Communication	4G LTE, 5G private networks, DSRC	4G deployed; 5G emerging	Low-latency data sharing; remote oversight	Bandwidth limits for HD point cloud sharing; coverage in pit walls
Infrastructure Sensing	Fixed cameras, radar at berms/dumps	Pilot deployments	Extended situational awareness beyond onboard sensors	No standardized integration protocol with AHS perception
Digital Twin Platforms	Hexagon, Trimble, Bentley iTwin	Operational in some tier-1 mines	Real-time pit model; planning support; simulation	Latency of twin update; feedback to vehicle perception pipeline
Geotechnical Monitoring	Slope radar (GroundProbe), MEMS, InSAR	Widely adopted for hazard warning	Bench stability data; subsidence mapping	Not integrated with the AHS safety envelope; no real-time trigger
Cooperative/Shared Perception	V2V raw or feature sharing (research)	Research prototypes only	Occlusion resolution; extended detection range	No production deployment; bandwidth; latency; trust

Note: The “Current Deployment Maturity” ratings presented in Table 3 are based on evidence reported in peer-reviewed publications and industry reports reviewed in Section 5.1, Section 5.2, Section 5.3, Section 5.4 and Section 5.5. These ratings do not represent independent verification by the authors of the deployment status at any specific mine site. Similarly, the “Key Research Gaps” identified in the table are derived from limitations explicitly discussed in the cited literature and, where applicable, from the absence of documented evidence of system integration, validation, or large-scale operational deployment in the reviewed sources.

**Table 6:** ECDV framework; five-layer architecture with components, data sources, functions, and inter-layer outputs.

ECDV Layer	Primary Components	Data Sources	Key Functions	Output to Next Layer
L1: Onboard Perception	LiDAR, camera, radar, GNSS/IMU	Vehicle sensors	Real-time object detection, segmentation, and ego-motion estimation	Local occupancy map; object list; ego-state vector
L2: Cooperative Perception	V2X links; edge servers; RSU cameras	Multi-vehicle infrastructure	Shared BEV map construction; occlusion filling; conflict-zone awareness	Extended fused occupancy map; shared hazard layer
L3: Ecosystem Context Layer	Digital twin; FMS; geotechnical feeds	Minewide databases; monitoring platforms	Terrain state; slope risk index; traffic intent; blast schedule	Risk-annotated environment model; intent-aware route
L4: Predictive Safety and Planning	ML hazard models; probabilistic planners	L1–L3 fused data	Predictive hazard modeling, risk-aware path planning, and proactive speed management	Safe velocity profile; hazard alerts; maintenance triggers
L5: Human–Machine Interface	Control room dashboards; operator alerts	L4 outputs	Situation display; intervention requests; audit logging	Operator decisions; system overrides; regulatory records

## Data Availability

No new data were created or analyzed in this study. Data sharing does not apply to this study.

## Abbreviations

The following abbreviations are used in this manuscript:

**Acronym**	**Definition**
AHS	Autonomous Haulage System
AI	Artificial Intelligence
BEV	Bird’s Eye View
C-V2X	Cellular Vehicle-to-Everything
dB	Decibels
DSRC	Dedicated Short-Range Communications
ECDV	Ecosystem-Centric Dynamic Vision
FMS	Fleet Management System
FP32	32-bit Floating Point
FPGA	Field-Programmable Gate Array
GHz	Gigahertz
GNSS	Global Navigation Satellite System
HD	High Definition
HD Map	High-Definition Map
IMU	Inertial Measurement Unit
InSAR	Interferometric Synthetic Aperture Radar
IoT	Internet of Things
IoU	Intersection over Union
ISO	International Organization for Standardization
KD	Knowledge Distillation
LiDAR	Light Detection and Ranging
mAP	Mean Average Precision
MEMS	Micro-Electro-Mechanical Systems
MEC	Mobile Edge Computing
mbps	Megabits per second
ms	Millisecond
NDS	NuScenes Detection Score
OSF	**Open Science Framework**
PRISMA	Preferred Reporting Items for Systematic Reviews and Meta-Analyses
PTQ	Post-Training Quantization
R-CNN	Regions with Convolutional Neural Networks
RSU	Road-Side Unit
SAE	Society of Automotive Engineers
SAM	Segment Anything Model
SLAM	Simultaneous Localization and Mapping
TASP	Trigger Action Response Plan
VLM	Vision-Language Model
V2I	Vehicle-to-Infrastructure
V2N	Vehicle-to-Network
V2P	Vehicle-to-Pedestrian
V2V	Vehicle-to-Vehicle
V2X	Vehicle-to-Everything
ViT	Vision Transformer
YOLO	You Only Look Once
